# Grubs in the Face—Acne Punctata

**Published:** 1867-10

**Authors:** 


The following excerpta we make from the Druggists' Circular:

### *Grubs in the Face—Acne Punctata.*

It is no easy thing to remove this affection from the face, for it seems to be dependent, in a great measure, on the physiological functions of the sebaceous follicles of the skin, which are peculiarly developed in persons suffering from this unsightly eruption. The grub-like matter is the secretion of the follicle, thickened like cheese, and confined by an obstruction of the orifice. The black head is only dirt at the mouth of the follicle, adherent to the sabaceous matter. It is said there is actually an animalcule in each little cast, that may be pressed out by squeezing, and it has been termed *Steatozoon folliculorum*. Remedies seem to have but little effect in curing acne, for there is in truth no particular disease to remove. Stimulants, or whatever excites the cutaneous circulation, should be avoided—the bowels kept moderately open, with some one of the mineral purgatives, and, instead of soap, a solution of twenty grains of the carbonate of soda dissolved in a quart of water, may be used as a detergent. A spirituous solution, consisting of two drachms of oil of lemon and half a drachm of oil of rosemary in a pint of alcohol, should be applied as a lotion to the skin immediately after washing with the soda water.

In chronic cases, to the foregoing lotion may be added advantageously half a grain of corrosive sublimate to each ounce of the spirit.

